# The Effect of CO_2_ on Algal Growth in Industrial Waste Water for Bioenergy and Bioremediation Applications

**DOI:** 10.1371/journal.pone.0081631

**Published:** 2013-11-22

**Authors:** David A. Roberts, Rocky de Nys, Nicholas A. Paul

**Affiliations:** School of Marine and Tropical Biology, James Cook University, Townsville, Australia; University of New South Wales, Australia

## Abstract

The energy, mining and mineral processing industries are point sources of metal-contaminated waste water and carbon dioxide (CO_2_). Freshwater macroalgae from the genus *Oedogonium* can be grown in metal-contaminated waste water to generate biomass for bioenergy applications and concomitantly bioremediate metals. However, interactions between CO_2_ addition and algal growth, which can affect bioremediation, remain untested. The addition of CO_2_ to algal cultures in the Ash Dam Water (ADW) from a coal-fired power station increased the biomass productivity of *Oedogonium* sp. from 6.8 g dry weight (DW) m^-2^ d^-1^ to a maximum of 22.5 g DW m^-2^ d^-1^. The greater productivity increased the rate of bioremediation of most elements. However, over time carbon-amended cultures experienced a decline in productivity. Possible explanations include metal toxicity at low pH or essential trace element limitation as a result of competition between toxic and essential trace elements for uptake into algae. Higher productivity increased bioremediation rate and yielded more biomass for bioenergy applications, making maintenance of maximum productivity the central aim of the integrated culture model. To do so it will be necessary to resolve the mechanisms responsible for declining yields over time in carbon-amended cultures. Regardless, our data demonstrate that freshwater macroalgae are ideal candidates for bioremediation of metal-contaminated waste streams. Algal culture delivered significant improvement in ADW quality, reducing 5 elements that were initially in excess of water quality criteria (Al, As, Cd, Ni and Zn) to meet guidelines within two to four weeks.

## Introduction

A barrier to the development of renewable biofuels is the production of biomass in a manner that does not conflict with simultaneous demands on arable land for food production [1]. There is intense interest in the development of biofuels from macroalgae (hereafter algae) that can be cultivated on non-arable land [2,3]. The use of algae as a feedstock for bioenergy applications is not, however, without its own challenges. Two of the main costs and barriers to sustainability incurred in algal production are a source of large quantities of suitable water for cultivation and inorganic carbon (CO_2_) [4–6]. The use of large quantities of potable water to produce algae is unsustainable, particularly in arid regions that often have the most amenable climates for algal production [7], and this issue will only intensify as aridity becomes more widespread with climate change [8]. Similarly, if large-scale intensive algal cultivation is to become a reality then sustainable and cost effective CO_2_ sources must be identified. The dissolution of atmospheric carbon into water is limited by the high surface tension of water and relatively low CO_2_ content of the atmosphere and this leads to carbon limitation in intensive algal culture systems [4,9]. As the addition of supplementary CO_2_ can double productivity, it is a critical step towards achieving the high biomass requirements to support a future biofuels industry [10,11], yet the high cost of commercial carbon sources is a barrier to cost-effective algal biofuels [5].

One model that overcomes these two key barriers is to integrate algal culture with industries that produce non-potable waste water and waste inorganic carbon (CO_2_). It has long been recognized, for example, that algae can be cultivated in nutrient and organic-rich waste water from agriculture, aquaculture [12–15] and municipal waste water [16] to remediate excess nutrients from effluents. It has also been demonstrated at laboratory scales that algae can be cultured in metal-contaminated industrial effluents and accumulate dissolved metal ions from waste water in the process [17–19]. The energy, mining and mineral processing industries have point source emissions of waste water with high levels of metals and CO_2_, providing a platform to generate biomass for bioenergy applications using integrated algal culture with simultaneous metal bioremediation and carbon capture. Water and inorganic carbon can be provided by the industrial facility in the form of waste water and flue gas respectively, potentially in exchange for metal bioremediation (water re-use options) and carbon capture services.

The success of the coal-fired power station model for integrated algal culture relies upon an ability to produce biomass in marginal waste water while simultaneously sequestering metals and capturing carbon. Small scale laboratory experiments have shown it is possible to cultivate freshwater algae in these metal-contaminated waste streams with the addition of limiting macronutrients such as N and P [19]. However, key gaps remain in our understanding of the potential to intensively produce biomass in metal-contaminated waste water. In the simplest sense, algal production for the purposes of generating biofuel feedstock should maximize productivity through the addition of CO_2_ to cultures. In turn, the greater productivity should lead to greater metal bioremediation through the production of metal binding sites on the freshly grown algae. However, the effects of CO_2_ on growth and subsequent bioremediation cannot be considered in isolation as CO_2_ addition will also alter the pH of waste water and therefore the relative concentration and consequently bioavailability of free metal ions. Any increases in the concentrations of free metal ions could result in greater metal uptake by algae (and hence improved bioremediation), but could equally carry an increased risk of toxicity and negative effects on biomass production [20]. Finally, as some elements are more sensitive than others to shifts in pH, CO_2_ addition may favour the accumulation of some elements by algae, while accumulation of other elements remains unchanged. Resolving the interactions between these processes is critical for the integrated culture of algae to deliver simultaneous metal bioremediation and carbon capture while producing biomass for bioenergy applications.

We have previously demonstrated that the multi-element waste water from coal-fired power generation is a suitable culture media for the freshwater alga *Oedogonium* sp. under laboratory conditions [19]. In this study we conduct experiments with the same *Oedogonium* sp. in the same effluent to examine the effect of CO_2_ addition on algal productivity and metal bioremediation in open outdoor cultures. We move beyond a controlled laboratory environment to an intermediate-scale open outdoor culture system where temperature and light vary naturally with no external control. We address two key questions. First, what are the effects of CO_2_ on productivity of *Oedogonium* sp. in metal-contaminated waste water? Second, how does productivity relate to the rate of bioremediation through time?

## Materials and Methods

### Experimental facility and algae production

Experiments were conducted in Ash Dam Water (ADW) from a 1400 megawatt coal-fired power station (Tarong) in Queensland, Australia (19.33°S, 146.76°E). The flue stacks at Tarong are washed down with freshwater to remove residual ash. This water contains a high density of ash and a range of metals (e.g. Al, Cd, Mo, Ni, V and Zn) and metalloids (e.g. As, B and Se) in excess of Australian water quality criteria (Table 1) [21]. All ADW is retained on site and this has necessitated heightening of the dam wall to accommodate seasonal peaks in rainfall [22]. ADW was collected from Tarong power station with the permission of Stanwell Corporation Ltd in October 2012 and transported to James Cook University (JCU), Townsville, in 1000L intermediate bulk carriers (IBCs), then stored at the Marine and Aquaculture Research Facilities Unit (MARFU) at JCU.

**Table 1:** Concentration of metals, metalloids and other elements in Dechlorinated Town Water and Ash Dam Water.

**Element**	**Units**	**Dechlorinated Town Water**	**Ash Dam Water**	**ANZECC Trigger Value (95% protection)**
**Al**	µg L^-1^	<LOD	**110 ± 23**	55
**As**	µg L^-1^	<LOD	**33.5 ± 3**	24
**B**	µg L^-1^	<LOD	**4650 ± 95**	370
**Cd**	µg L^-1^	<LOD	**0.6 ± 0.1**	0.2
**Cr**	µg L^-1^	<LOD	<LOD	1.0
**Cu**	µg L^-1^	0.8 ± 0.1	1.0 ± 0.5	1.4
**Fe**	µg L^-1^	<LOD	27.5 ± 2.6	ID
**Pb**	µg L^-1^	<LOD	<LOD	3.4
**Mn**	µg L^-1^	0.6 ± 0.1	5.5 ± 5	1900
**Hg**	µg L^-1^	<LOD	<LOD	0.6
**Mo**	µg L^-1^	<LOD	1103 ± 61	ID
**Ni**	µg L^-1^	<LOD	**29.8 ± 1**	11
**Se**	µg L^-1^	<LOD	**42.5 ± 3**	11
**SrSr**	µg L^-1^	54.5 ± 1.00	2078 ± 25	ID
**V**	µg L^-1^	<LOD	1058 ± 11	ID
**Zn**	µg L^-1^	<LOD	**52.5 ± 11**	8.0
**Ca**	mg L^-1^	8.0 ± 0.01	338.5 ± 2.33	ID
**Na**	mg L^-1^	15 ± 0.01	468.8 ± 3.68	ID
**Mg**	mg L^-1^	2 ± 0.01	92.0 ± 0.58	ID
**K**	mg L^-1^	1 ± 0.01	44.3 ± 0.48	ID

Bold values exceed the ANZECC water quality criteria for freshwater bodies at the 95% protection level [21]

All data are mean concentrations (µg l^-1^) ± S. E. (*n* = 4)

LOD = Limit of Detection

ID = Indeterminate, no trigger value exists in ANZECC guidelines [21]

Experiments used the alga *Oedogonium*, which is a genus of unbranched filamentous algae. Freshwater macroalgae from the genus *Oedogonium* are cosmopolitan and grow attached to the substrate or as free floating mats. *Oedogonium* is also found in water bodies within the confines of Tarong power station. It is a competitively dominant macroalga that overgrows other algae under conditions of nutrient excess [23–25] and achieves high productivities in CO_2_-amended monocultures [10]. Our experiments used the same *Oedogonium* isolate originally used by Saunders et al. [19] and later by Lawton et al. [23]. The strain was not identified to species level given the lack of defining morphological characteristics and matching DNA sequences (see Lawton et al. [23] for genbank accession numbers). However, the strain is considered unique and is hereafter referred to as *Oedogonium* sp. The original biomass used in this experiment was collected from an irrigation channel in the Brandon region, Queensland, Australia (latitude: 19.55°S; longitude: 146.35°E) and a live stock culture is maintained in dechlorinated water with f/2 media [26] in a library collection at MARFU (19.33°S, 146.76°E).

### Experimental design

*Oedogonium* sp. was cultured under several carbon addition treatments in outdoor open culture systems to assess the effects of CO_2_ on growth and metal bioremediation by algae, and metal speciation in ADW. Environmental parameters (temperature and light) were not controlled as these factors would also be left to vary naturally under large-scale algal cultivation. The ADW contains many of the trace elements in traditional f/2 growth media (e.g. Cu, Fe, Mn and Mo). Therefore, only N and P were added as nutrients to determine productivity and bioremediation with minimal nutrient supplementation and to avoid unnecessary additions of trace elements to treated water. A series of control cultures in Dechlorinated Town Water (DTW) were included that received only N and P to evaluate the effect of trace element limitation on productivity in clean water cultures. N and P were added each week after the harvest to ensure N and P were not limiting during the experiment. All replicates were provided with N and P from NaNO_3_ and KH_2_PO_4_ stock solutions respectively at concentrations equivalent to f/2 (12.5 mg l^-1^ N and 1.3 mg l^-1^ P).

Algae were cultured in ADW and DTW under three treatments (no CO_2_, 3L min^-1^ CO_2_ and 6L min^-1^ CO_2_) with 4 replicate tanks per treatment. Each replicate was a 60L plastic tank stocked with *Oedogonium* sp. at a density of 0.5 g fresh weight (FW) L^-1^. The carbon-amended treatments were dosed with CO_2_ (food grade 99.9% BOC) for 20 sec every 10 min at the respective gas flow rates between 8:00 and 16:00 each day. Given regulations in Australia regarding the transport of bottled flue gas, and the fact that experiments were conducted at JCU, it was necessary to use commercial CO_2_ for these experiments. The CO_2_ pulses were controlled by a solenoid and digital timer, with CO_2_ delivered to tanks through a manifold airline with aquarium air stones. Tanks were aerated to keep the filaments in suspension. The pH was recorded in each tank at 09:00, 12:00 and 15:00 each day.

Biomass was harvested every 7 d for 4 weeks, spun to a constant weight then weighed (nearest 0.1 g FW). Each tank was restocked with a 30 g sub-sample of the harvested biomass to re-set the density at 0.5 g FW L^-1^ and N and P added to ensure they were not limiting. Evaporative loss was replaced with DTW. The surplus biomass was dried at 60°C for 48 h and growth converted to areal productivity (g dry weight [DW] m^-2^ d^-1^). Biomass samples were taken at weekly intervals for elemental analysis. Diffusive Gradients in Thin Films (DGTs) were deployed in the middle of the tanks with nylon fishing line and retrieved after 72 h deployment time to measure the bioavailable fraction of elements [27]. The Chelex® resin was extracted and the elements eluted with 1ml of ultrapure 1M HNO_3_ for 24 h. The acid extract was diluted with deionized water and analysed as described below.

### Elemental analysis

Dried biomass cultured in ADW was analyzed for 20 trace elements. 100 milligrams (mg) of the dried algae was placed in a Teflon digestion vessel with 3.0 milliliters (ml) double distilled HNO_3_ and 1.0 ml analytical grade H_2_O_2_. The solution was digested for 2 h then heated in a microwave oven to 180°C for 10 min, then diluted with Milli-Q water. Major elements (Ca, K, Na and P) were analysed by Inductively Couple Plasma Optical Emission Spectrometer (ICP-OES), while metals and metalloids (Al, As, B, Cd, Cr, Cu, Fe, Mg, Mn, Mo, Ni, Se, Sr, V and Zn) were analysed by ICP Mass Spectrometer (ICP-MS). The elements eluted from the DGT sampling devices (Al, Cd, Cr, Cu, Fe, Mg, Mn, Mo, Ni, Pb, Sr, V and Zn) were also analysed with ICP-MS. The DGT resin has a low affinity for oxyanions [28] so As and Se were less than Limits of Detection (LOD) in all DGT units. All analyses were conducted by the Advanced Analytical Center at JCU.

### Data analyses

Throughout this study we have quantified bioremediation as *in toto* element sequestration, which potentially includes both metabolically active internalization of elements into algal cells and passive adsorption of elements to the cell surface. We use this metric for two key reasons. First, both passive biosorption and metabolic internalization of elements are valid remediation strategies. Second, our method of processing biomass (harvesting followed by direct drying without rinsing) simulates an industrial process where post-harvest processing would use the minimal number of steps possible to produce a suitable feedstock for bioenergy production. Element bioremediation rates were derived from the product of the element concentrations in biomass (mg kg^-1^) and the areal productivity each week (g DW m^-2^ d^-1^), then converted to mg element m^-2^ d^-1^. Mass balance calculations were also performed to determine the proportion of reduction in aqueous elements that was accounted for by *in toto* algal sequestration.

Areal productivity and bioremediation by algae were analysed by Repeated Measures Analysis of Variance (ANOVA). For productivity, these analyses included the fixed between-subjects factors “water source” and “CO_2_”, and “time” as the within subjects factor. For element concentrations and bioremediation rates, only ADW biomass was considered so the factors were “CO_2_” and “time”. Normality and homogeneity of variance were examined with residual histograms and scatterplots of residuals vs. estimates respectively and data were transformed when necessary [29]. For “time” and its interactions with between-subjects effects, sphericity was determined by Huynh-Feldt epsilon values [29]. The elemental composition of biomass was also examined using Principal Components Analysis with varimax rotation. The concentration of elements accumulated by the DGT units was calculated according to published methods [27]. Tukeys post-hoc comparisons tested differences amongst means when significant interactions were detected. All statistical analyses were performed in SPSS version 20 and R [30].

## Results

### Effect of CO_2_ on productivity in ADW and DTW

**Figure 1:**
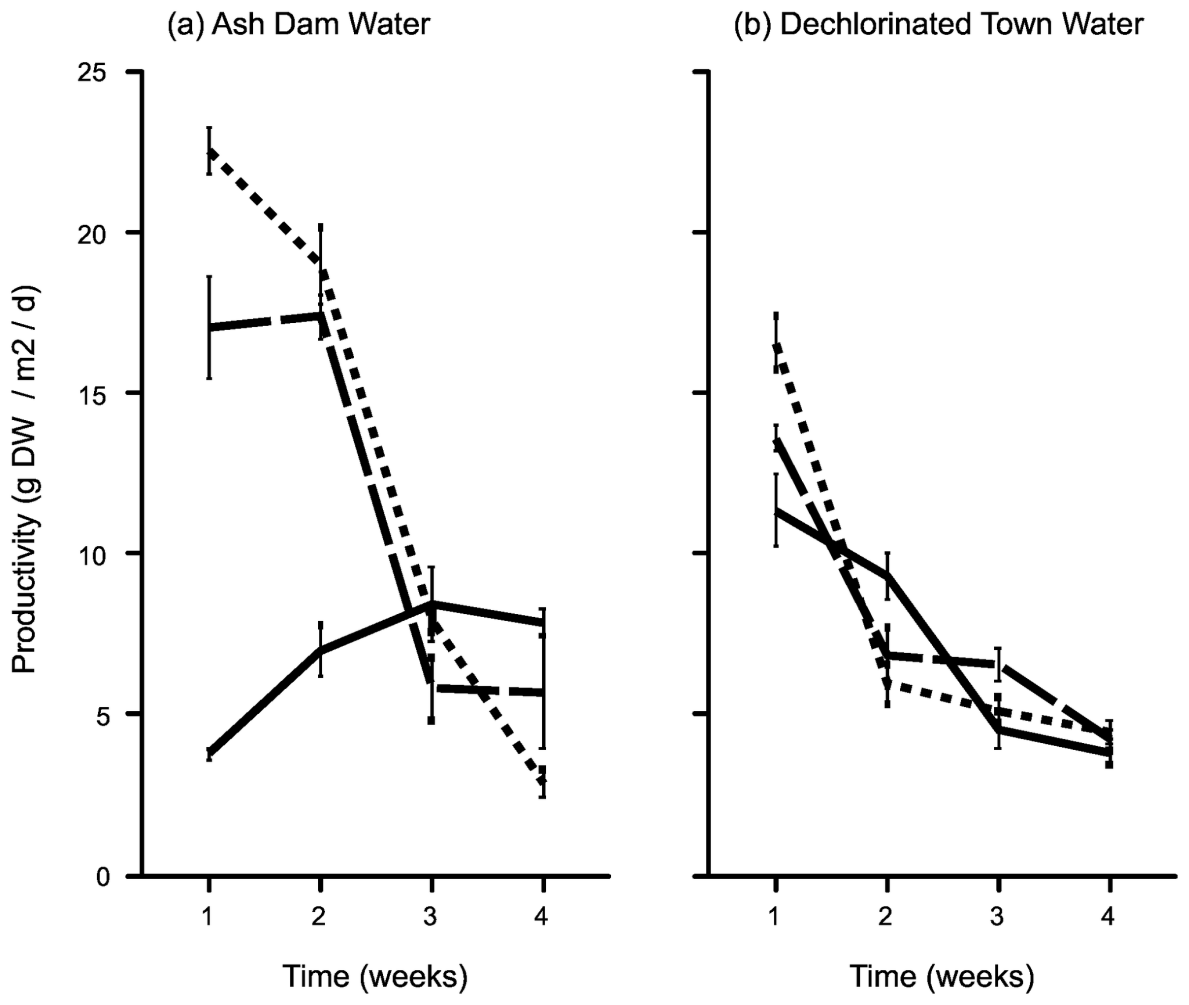
Areal productivity of *Oedogonium* sp. (g DW m^-2^ d^-1^) in a) Ash Dam Water and b) Dechlorinated Town Water during the four week culture experiments. Unbroken, dashed and dotted lines show data for the 0, 3 and 6 L min^-1^ CO_2_ treatments respectively.

The effects of CO_2_ addition on productivity of *Oedogonium* sp. varied through time in ADW and DTW (Figure 1, Time x WS x CO_2_; F_6,54_ = 24.83, P < 0.001). The addition of CO_2_ enhanced productivity of *Oedogonium* sp. in ADW, yielding 22.5 and 19.0 g DW m^-2^ d^-1^ in the 6L min^-1^ CO_2_ treatment in weeks one and two respectively. The yields were slightly lower in the 3L min^-1^ CO_2_ treatment (17 g DW m^-2^ d^-1^ after weeks one and two) and both were greater than the no CO_2_ treatment (<5 g DW m^-2^ d^-1^, Figure 1a). Productivity in the ADW cultures amended with CO_2_ declined after week two to mean growth rates of 3-5 g DW m^-2^ d^-1^ by week four (Figure 1a). In contrast, productivity in the no CO_2_ treatment in ADW increased from 3 to 8 g DW m^-2^ d^-1^ to become the treatment with the highest productivity (Figure 1a).

Productivity in the DTW cultures was initially 13.5 and 16.5 g DW m^-2^ d^-1^ in the 3 and 6L min^-1^ CO_2_ treatments, and 12 g DW m^-2^ d^-1^ in the no CO_2_ control (Figure 1b). Therefore growth of *Oedogonium* sp. in ADW with CO_2_ addition exceeded growth in the corresponding DTW treatments in weeks one and two (Figure 1a-b). Conversely, growth of *Oedogonium* sp. in ADW without CO_2_ addition was slower than the corresponding DTW treatment in weeks one and two (Figure 1a-b). After two weeks the yields in all treatments had reduced to <10 g DW m^-2^ d^-1^, converging at approximately 5 g DW m^-2^ d^-1^ by week four (Figure 1b), which were similar to the ADW cultures with CO_2_ addition, and lower than growth in the no CO_2_ ADW cultures (Figure 1a-b).

Maximum pH occurred at 15:00 each day, and averaged 9.91 ±0.04, 8.02 ± 0.06 and 7.26 ± 0.05 for no CO_2_, 3 and 6L min^-1^ CO_2_ treatments in ADW respectively. The respective DTW treatments had mean pH at 15:00 of 10.26 ± 0.05, 8.22 ±0.09 and 7.55 ±0.04. Average mean daily temperatures in the cultures were 27.2 ± 0.2°C during the experiment, with daily mean temperature ranging from 24.9 - 28.3°C. The mean weekly total photosynthetically active radiation (PAR) irradiance inside the greenhouse during the four week experimental period was 129.69 mol photons m^-2^ at the surface of the cultures. The peak daily irradiance was on average 1501 µmol photons m^-2^ s^-1^.

### Effect of CO_2_ on element bioavailability

**Figure 2:**
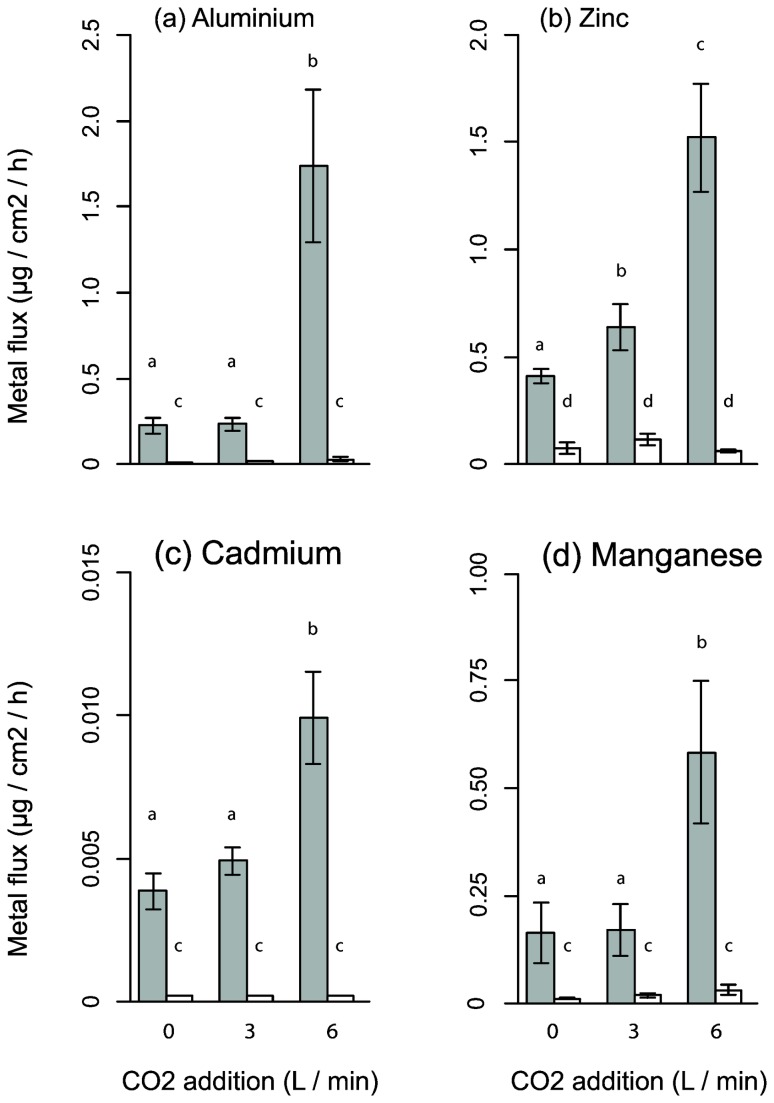
Metal flux from Ash Dam Water (grey bars) and Dechlorinated Town Water (white bars) under different CO_2_ amendment regimes. Bars sharing a common letter do not differ significantly (Tukey HSD P > 0.05).

The bioavailability of Al, Cd, Mn and Zn were all pH-dependent with 3-5 fold increases in the labile fraction of these elements when CO_2_ was added to ADW at 6L min^-1^ (Figure 2, Table S1). The mean midday pH was 6.62 in the 6L min^-1^ CO_2_ treatment at the time the DGTs were deployed, compared to 9.13 in the no-CO_2_ treatments. The flux of Al, Cd and Mn to labile states was higher at only the highest CO_2_ amendment rate, while Zn also showed a slightly increased flux at the intermediate rate of 3L min^-1^ CO_2_ (Figure 2). Cr, Fe and Ni were all unaffected by the CO_2_ treatments but were greater for the ADW than DTW (Table S1). Fluxes of Cu were typically low with no differences between the water sources or CO_2_ treatments (Table S1).

### Effect of CO_2_ on element uptake and concentration by *Oedogonium* sp. from ADW

**Figure 3:**
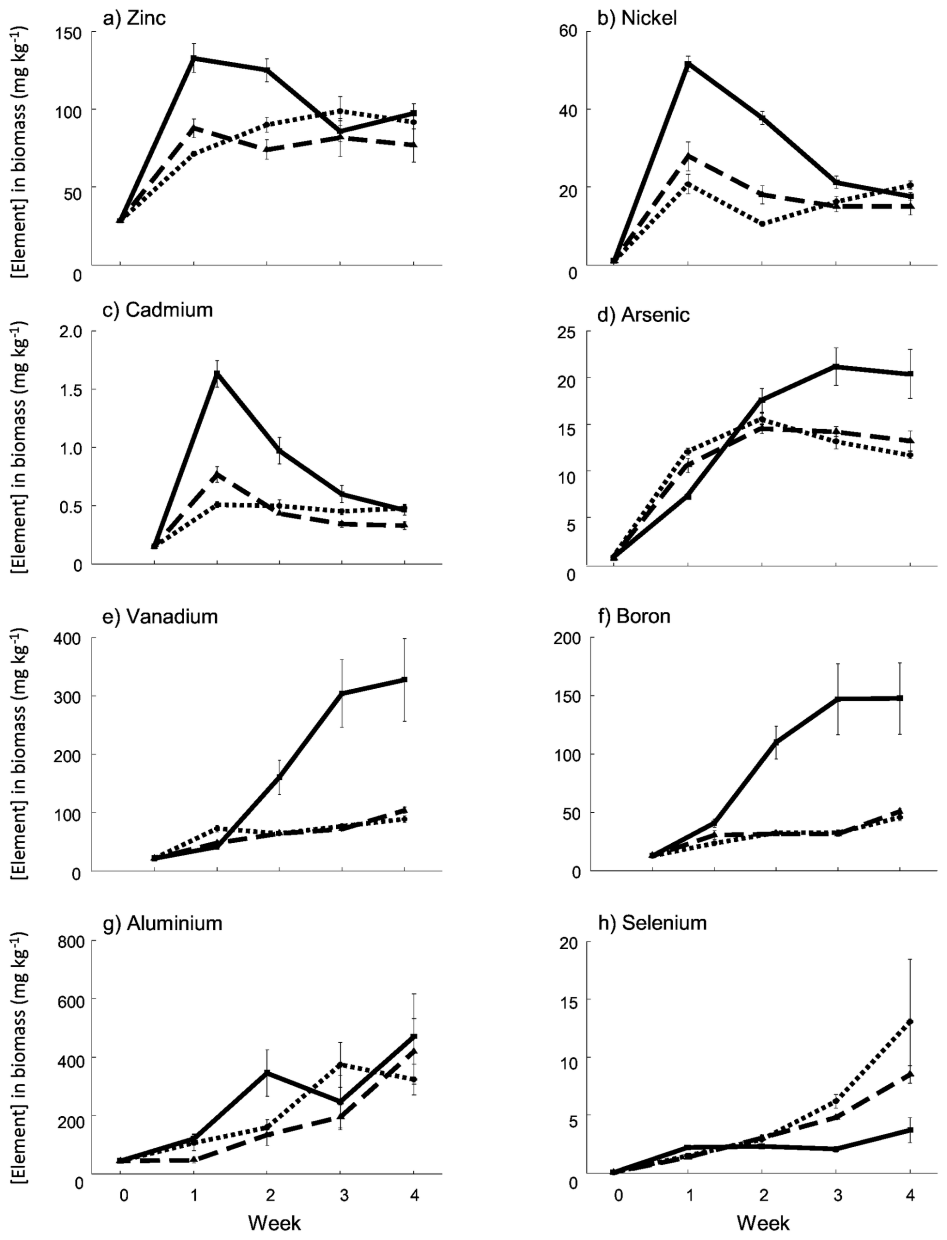
Metal and metalloid concentrations in *Oedogonium* sp. biomass cultured in Ash Dam Water at three levels of carbon supplementation. Unbroken, dashed and dotted lines show data for the 0, 3 and 6 L min^-1^ CO_2_ treatments respectively.

The effects of CO_2_ addition on the bioavailability of elements were not clearly conveyed in the elemental contents of the algae. Rather, CO_2_ had variable effects on the elemental composition of *Oedogonium* sp. across the experiment. The concentrations of most of elements increased in *Oedogonium* sp. biomass relative to concentrations at the start of the experiment, unequivocally showing element removal from the ADW (Week 0, Figure 3a-h). The sum of the elements in biomass taken from stock cultures was 0.31%, increasing more than four-fold to 1.30% in biomass from the no CO_2_ ADW cultures, while being unchanged at 0.31 and 0.25% in the 3L min^-1^ and 6L min^-1^ CO_2_ treatments respectively.

While in weeks one and two (“new” ADW) there were distinct differences between the elemental composition of *Oedogonium* sp. in treatments receiving CO_2_ relative to those with no CO_2_ addition, over time the concentrations tended towards a similar composition in all treatments (Figure S1). For most elements there was a “time x CO_2_^”^ interaction in the univariate analyses (Table S2). Biomass cultured without CO_2_ had higher concentrations of several elements in weeks one and two (particularly Zn, Ni and Cd), followed by declining concentrations until week four (Figure 3a-c). In contrast, As, V and B concentrations increased in biomass from the no CO_2_ treatment during weeks two to four (Figure 3d-f). There was no clear effect of CO_2_ addition on Al uptake (Figure 3g), while concentrations of Se were greatest in the CO_2_ amended treatments by the end of the experiment (Figure 3h).

**Figure 4:**
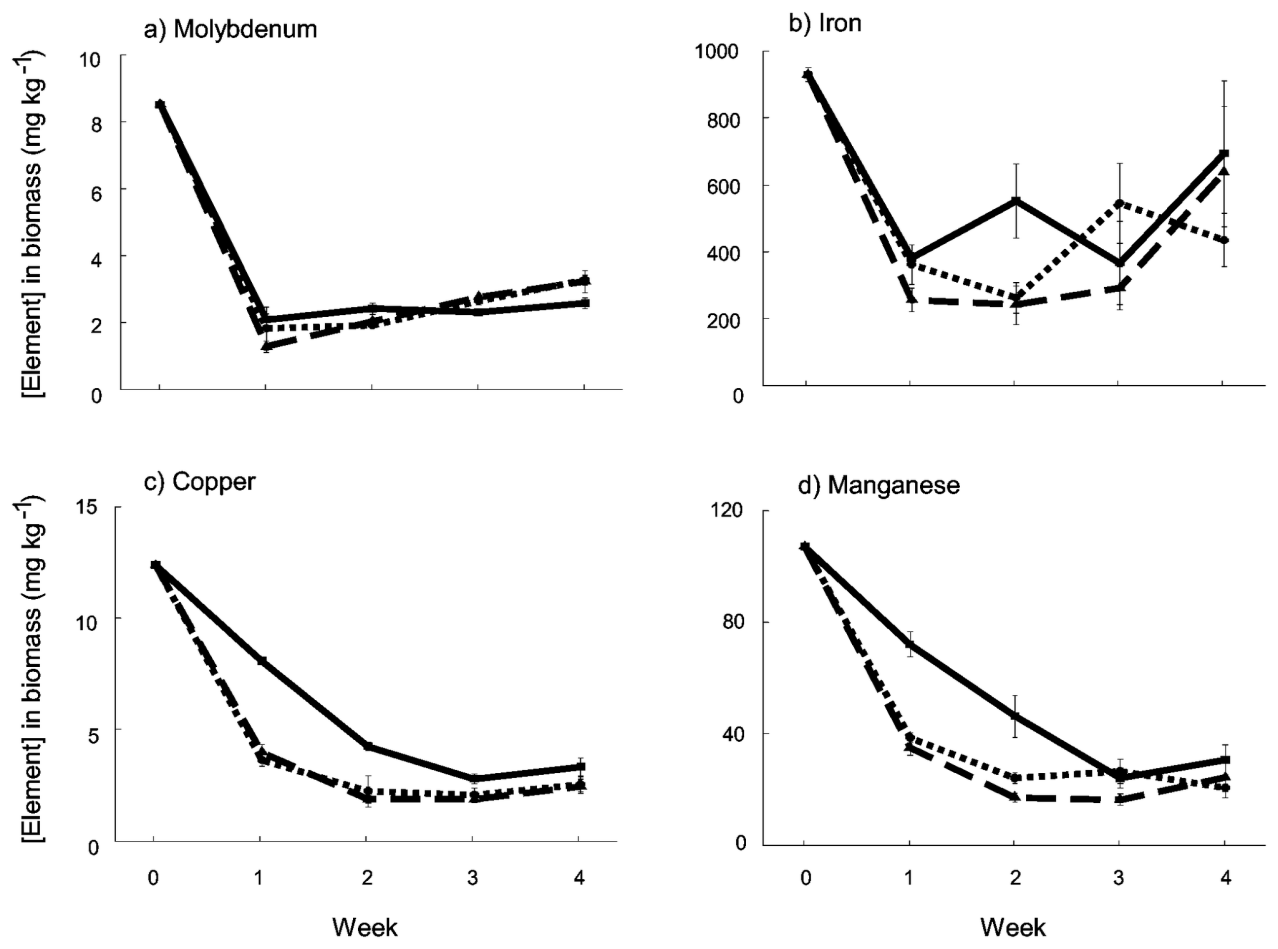
Concentrations of essential trace elements in *Oedogonium* sp. biomass cultured in Ash Dam Water at three levels of carbon addition. Unbroken, dashed and dotted lines show data for the 0, 3 and 6 L min^-1^ CO_2_ treatments respectively.

Several key trace elements decreased in *Oedogonium* sp. when cultured in ADW relative to concentrations in biomass from f/2 stock cultures (week 0, Figure 4a-d). These included Mo, Fe, Cu and Mn (Figure 4a-d). For Cu and Mn, these decreases in internal concentrations were most rapid in the CO_2_ amended treatments, while for Mo concentrations decreased rapidly in all treatments (Figure 4).

### Effects of CO_2_ and productivity on the rate of element removal from ADW

**Figure 5:**
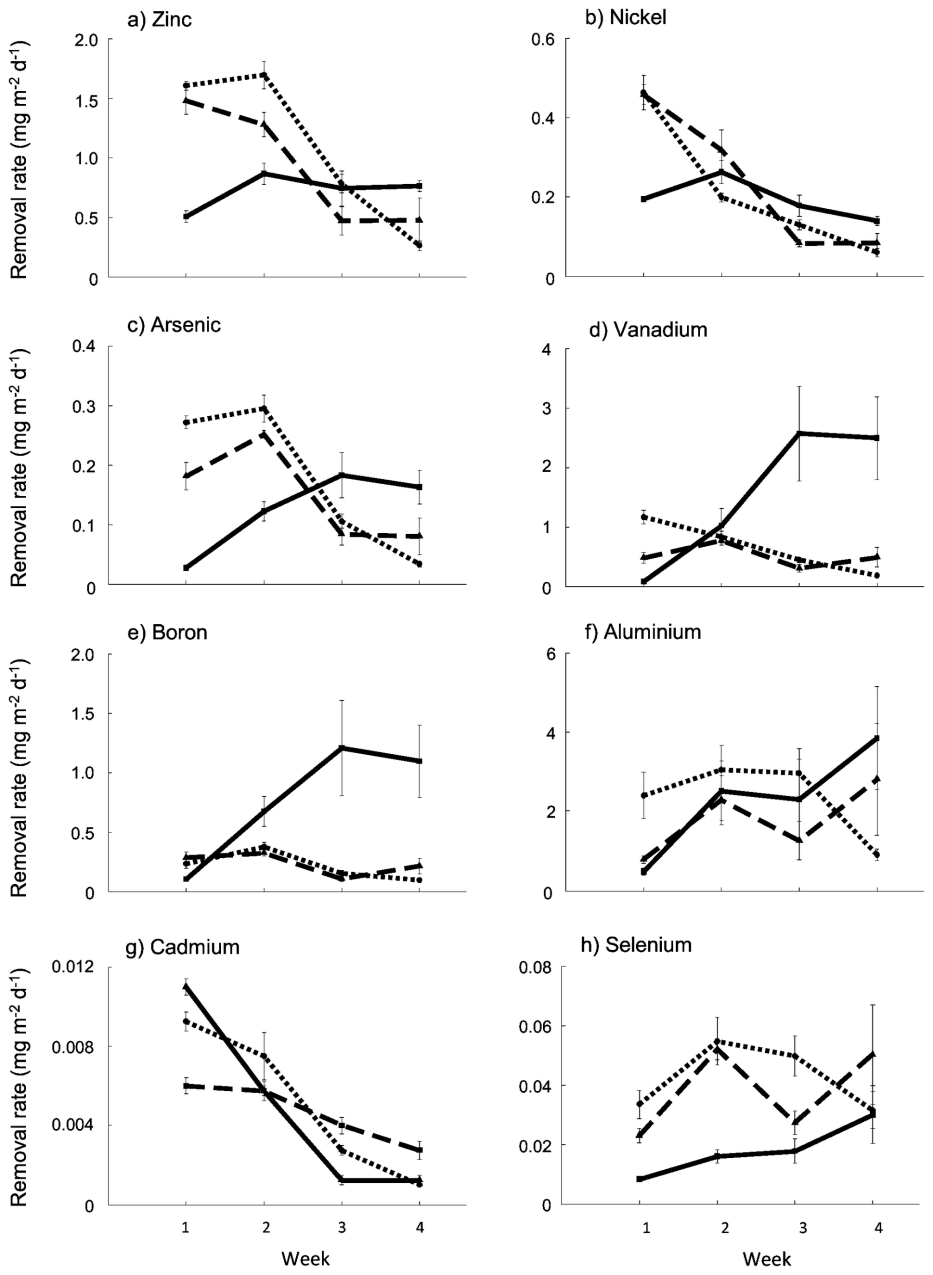
Areal sequestration (mg m^-2^ d^-1^) of metals and metalloids by *Oedogonium* sp. cultured in Ash Dam Water at three levels of carbon supplementation. Unbroken, dashed and dotted lines show data for the 0, 3 and 6 L min^-1^ CO_2_ treatments respectively.

As for raw biomass concentrations, there was no consistent effect of CO_2_ addition on element removal rates (Figure 5). Rather, the rate of removal of each element was a factor of productivity (g DW m^-2^ d^-1^) and element concentrations (mg kg^-1^). Element removal rate varied through time in each of the treatments (Table S3), with the rate of bioremediation tending to be greatest in the treatments with the highest productivity at each point in time (Figure 5). This was true even when total element concentrations in biomass from those treatments were lower. For elements with an apparently high affinity and therefore rapid accumulation in biomass (e.g. Zn, Ni and As), the highest overall removal rates were achieved in weeks one and two when CO_2_ addition increased productivity (Figure 5a-c). However, in weeks three and four, when productivity in these treatments declined, element removal rates were greatest in the no CO_2_ treatment (Figure 5a-c). Elements with a lower affinity for uptake (V, B, Al and Se) showed greater bioaccumulation in the later stages of the experiment. In week one, removal rates of V, B and Al were greatest in the carbon-amended treatments, however, the maximum removal rates were achieved in the no CO_2_ treatments in the later stages of the experiment (Figure 5d-f). Furthermore, it was notable for B and V that in weeks three and four the removal rates in the no CO_2_ treatment far outweighed removal in carbon-amended treatments during weeks one and two, despite the large disparity in productivities. Overall, 5 of the 7 elements in the ADW that initially exceeded ANZECC guidelines were reduced to below ANZECC levels (Al, As, Cd, Ni and Zn) during the culture period (Table 2). Mass balance calculations indicated that for each of these elements >75% of the reductions in aqueous element concentrations (and in most cases >95%) were accounted for by the element mass accumulated by *Oedogonium* sp. over the culture period.

**Table 2:** Concentration of metals and metalloids in Ash Dam Water before and after the four week experiment under different CO_2_ addition regimes.

**Element**	**Initial**	**Final (after 4 weeks of culturing)**		
		**No CO_2_**	**3L min^-1^ CO_2_**	**6L min^-1^ CO_2_**
**Al**	**110 ± 23**	13 ± 0.42	32.5 ± 9.52	53 ± 11.63
**As**	**33.5 ± 3**	16 ± 0.23	16.3 ± 2.13	17.5 ± 8.12
**B**	**4650 ± 95**	**4438 ± 51**	**4300 ± 109**	**4355 ± 17**
**Cd**	**0.6 ± 0.1**	<LOD4	<LOD4	<LOD4
**Ni**	**29.8 ± 1**	4.5 ± 0.13	4.8 ± 0.93	9.8 ± 0.83
**Se**	**42.5 ± 3**	**45.0 ± 3**	**40 ± 0**	**35 ± 5**
**Zn**	**52.5 ± 11**	8.0 ± 1.02	**10.8 ± 0**.**8**2	**10.0 ± 0**.**7**2

Bold values exceed the ANZECC water quality criteria for natural freshwater bodies at the 95% protection level [21]

All data are mean concentrations (µg L^-1^) ± S. E. (*n* = 4)

Superscript numbers indicate the week in which ADW concentrations first fell below ANZECC trigger values (Table 1).

## Discussion

Through this study we demonstrate that it is possible to produce biomass for bioenergy applications in the marginal waste-water from coal-fired power generation while simultaneously providing significant improvements in water quality and carbon capture. The sum of the bioremediation over the 4 weeks of culture resulted in 5 of the 7 elements in the ADW that initially exceeded ANZECC guidelines being reduced to below ANZECC levels (Al, As, Cd, Ni and Zn). Furthermore, the greatest rates of bioremediation from ADW were achieved when productivity was greatest under conditions of carbon addition. For elements that are rapidly accumulated by algae, increasing biomass productivity is therefore the most direct means of increasing element bioremediation rates. High productivity of biomass with low element content is a more effective bioremediation strategy than low productivity of biomass with high element content. These results are in agreement with previous studies that have shown bioconcentration of most elements from ADW is positively correlated with growth rate [19]. Therefore, in the short-term, the addition of CO_2_ has dual benefits in increasing productivity of lower element content biomass for bioenergy applications and increasing the overall rate of element removal from waste waters.

In the longer-term we found biomass production to decrease in carbon-amended cultures with the effect of also reducing element bioremediation rate. There are several possible mechanisms that may be responsible for this pattern. At low pH we found the concentration of bioavailable elements increased (particularly Al) and this increased bioavailability may have had a toxic effect [31]. The bioavailability of Al is extremely pH sensitive and, as such, Al is typically a major contributor to ecotoxicity observed in acidified lakes and streams [31]. While the increased availability of dissolved metal ions at low pH may be beneficial in the short-term with respect to uptake and bioremediation, in the long-term the result may be increased risk of toxicity. An alternative explanation for the declining productivities in carbon-amended cultures over time is trace element limitation. Algae require several trace elements (e.g. Fe, Mn, Cu and Mo) to support intensive production. While we have not specifically distinguished between intracellular and extracellular element contents, the *in toto* elemental analysis indicates the algae cultured in ADW had significantly lower concentrations of these essential trace elements than those from stock cultures in DTW. This pattern is somewhat surprising for Mo in particular, given the high concentrations in ADW (~1 mg l^-1^) relative to f/2 media (~2-4 µg l^-1^) [26]. The declining concentrations of essential trace elements in algae despite their apparent bioavailability in the effluent may indicate competition for uptake between other elements in the ADW. Elements such as Al have no known biological function [31] and were rapidly accumulated by biomass at low pH, potentially at the expense of essential trace elements.

The maintenance of high productivity and bioremediation rates in contaminated waste water may, therefore, require strategic carbon utilization strategies and the addition of essential trace elements beyond their bioavailability in effluents. Understanding the mechanism underlying the declining productivities in carbon-amended cultures will be an important step towards successful biomass production and remediation in contaminated waste waters. Regardless, while productivities varied through time between the CO_2_ treatments, productivity was always highest in one of the ADW treatments. The increased growth in ADW over time without CO_2_ addition also confirms the ability of *Oedogonium* sp. to acclimate to ADW [19]. Algae may acclimate to high metal concentrations in water through various mechanisms including the production of metal-binding phytochelatins [32] and polyphosphate bodies [33], or sequestration of metals in storage vacuoles [34]. Thus, while there is a need to understand the mechanism underlying the decreased growth of biomass over time in ADW with CO_2_ addition (i.e. at lower pH), ADW is potentially a more suitable water source for *Oedogonium* sp. culture than DTW despite the relatively high concentrations of some metals and metalloids.

Our results also highlight the bespoke nature of optimising productivity and bioremediation in industrial waste. As the metal and mineral profile of effluent will vary for each water source, site specific regimes of carbon utilization and trace element addition will also be required. Furthermore, we demonstrate that there is a sequence in element removal from complex waste streams, with a clear preference of algae for uptake of a specific suite of cationic elements. The overall response was rapid uptake of some elements (particularly Al, Zn, Ni and Cd), followed by a second phase of bioaccumulation where other elements were taken up (particularly As, V and B). B and V uptake is rapid in laboratory cultures with no CO_2_ addition, and increases with productivity [19]. Together, these results indicate V and B uptake is improved under conditions of no carbon addition once ions with high affinity for uptake, such as Ni and Zn, are removed. In terms of achieving ANZECC trigger values, bioremediation was most effective for cationic metals with comparatively little uptake of metalloids and oxyanionic elements (the only exception being As which eventually attained water concentrations below ANZECC trigger values). Indeed, some elements (e.g. B and V) were only accumulated after several weeks of exposure to ADW. Our results and those from marine bioremediation studies indicate that cultivation systems with relatively short contact times between waste water and algae will be effective for contaminants that are rapidly accumulated by algae, such as Zn and Ni [35,36].

Experiments to date on algal-based metal bioremediation have focused on the kinetics of biosorption by dead biomass from mock solutions containing one metal [36,37]. As a result, we have a limited ability to predict how elemental composition of waste water will affect *in situ* bioremediation with live algae until more empirical evidence for different effluents is available [36]. Given effluent characteristics will vary greatly from site to site algal cultivation strategies will need to be tailored for individual waste water bodies and to suit local regulatory requirements. Our data show that additional treatment of effluents will probably be required to achieve regulatory limits if high concentrations of metalloids (e.g. Se and B) are present as these have a slow rate of uptake into algae. Biochar derived from dried *Oedogonium* sp. biomass has a strong affinity for metalloids (unpublished data). As biochar is a by-product of the conversion of biomass to biofuel, one can therefore convert the waste from bioenergy applications into a biosorbent for the post-harvest polishing of metalloids from waste water. This is a highly novel closed-system where all materials required to remediate effluents are produced on-site as part of the bioenergy production process. Furthermore, while the integrated algal culture model has clear applications in conjunction with existing coal-fired power generation facilities, any future bioenergy industry will also produce contaminated waste water and release CO_2_ from the biomass conversion process [38]. It is clear, therefore, that the integrated algal culture model is not only of direct benefit in existing energy-production industries but will also be critical to the development of a sustainable bioenergy industry. Our model allows bioenergy feedstocks to be produced at energy production facilities in a truly sustainable manner.

## Supporting Information

Figure S1 **Element composition ordination of *Oedogonium* sp. cultured in Ash Dam Water at three levels of carbon addition.** The Principal Coordinates Analysis was performed on the dataset as a whole however; results are shown in separate panels for each week for clarity. Black, grey and white circles depict data for no CO_2_, and 3 and 6L min^-1^ CO_2_ treatments respectively.
(EPS)

Table S1 **Analyses of Variance of element flux to the Diffusive Gradient in Thin Film units under different CO_2_ addition regimes.** Significant main effects or interactions (P < 0.05) are highlighted in bold.
(DOCX)

Table S2 **Repeated Measures Analyses of Variance of element concentrations in *Oedogonium* sp. biomass cultured in Ash Dam Water under different CO_2_ addition regimes.** Significant main effects or interactions (P < 0.05) are highlighted in bold.
(DOCX)

Table S3 **Repeated Measures Analyses of Variance of element removal rates from Ash Dam Water under different CO_2_ addition regimes.** Significant main effects or interactions (P < 0.05) are highlighted in bold.
(DOCX)
