# Supplementary material for: The Effect of CO2 on Algal Growth in Industrial Waste Water for Bioenergy and Bioremediation Applications
Source: PLoS One. 2013 Nov 22;8(11):e81631. doi: 10.1371/journal.pone.0081631 (PMC3838398; doi:10.1371/journal.pone.0081631)
Supplement: Table S1 — Analyses of Variance of element flux to the Diffusive Gradient in Thin Film units under different CO2 addition regimes. Significant main effects or interactions (P < 0.05) are highlighted in bold. (DOCX) [file pone.0081631.s002.docx]

Supporting Information

Table S1

|  |  | Al^a^ | | Cd^a^ | | Cr | | Cu^a^ | | Fe | | Mn | | Ni | | Zn^a^ | |
| --- | --- | --- | --- | --- | --- | --- | --- | --- | --- | --- | --- | --- | --- | --- | --- | --- | --- |
| **Source** | **df** | **MS** | **F** | **MS** | **F** | **MS** | **F** | **MS** | **F** | **MS** | **F** | **MS** | **F** | **MS** | **F** | **MS** | **F** |
| Water source (WS) | 1 | 14.12 | 135.14 | 0.020 | 109.68 | 4.10E-05 | **13.36** | 0.001 | 0.36 | 0.194 | **11.52** | 0.49 | 20.21 | 3.093 | **424.80** | 13.84 | 215.41 |
| CO_2_ | 2 | 2.03 | 19.43 | 0.002 | 9.77 | 6.70E-07 | 0.22 | 0.002 | 0.76 | 0.003 | 0.17 | 0.12 | 5.13 | 0.011 | 1.57 | 0.57 | 8.83 |
| WS x CO_2_ | 2 | 1.45 | **13.91** | 0.002 | **9.77** | 8.17E-06 | 2.69 | 0.005 | 2.19 | 0.010 | 0.56 | 0.11 | **4.38** | 0.013 | 1.72 | 0.81 | **12.59** |
| Residual | 18 | 0.10 |  | <0.001 |  | 3.04E-06 |  | 0.002 |  | 0.017 |  | 0.02 |  | 0.007 |  | 0.06 |  |

^a^Log transformed
