# Supplementary material for: The Effect of CO2 on Algal Growth in Industrial Waste Water for Bioenergy and Bioremediation Applications
Source: PLoS One. 2013 Nov 22;8(11):e81631. doi: 10.1371/journal.pone.0081631 (PMC3838398; doi:10.1371/journal.pone.0081631)
Supplement: Table S2 — Repeated Measures Analyses of Variance of element concentrations in Oedogonium sp. biomass cultured in Ash Dam Water under different CO2 addition regimes. Significant main effects or interactions (P < 0.05) are highlighted in bold. (DOCX) [file pone.0081631.s003.docx]

Supporting Information

Table S2

|  |  | Al^a^ | | As^a^ | | B^a^ | | Cd^a^ | | Ni^a^ | | Se^a^ | | V^a^ | | Zn | |
| --- | --- | --- | --- | --- | --- | --- | --- | --- | --- | --- | --- | --- | --- | --- | --- | --- | --- |
| **Source** | **df** | **MS** | **F** | **MS** | **F** | **MS** | **F** | **MS** | **F** | **MS** | **F** | **MS** | **F** | **MS** | **F** | **MS** | **F** |
| **Between subjects** |  |  |  |  |  |  |  |  |  |  |  |  |  |  |  |  |  |
| CO_2_ | 2 | 0.205 | 2.95 | 0.023 | 4.91 | 1.926 | 126.65 | 0.462 | 26.77 | 0.292 | 84.88 | 0.242 | 7.85 | 0.767 | 51.64 | 3920.7 | 10.47 |
| Residual | 9 | 0.070 |  | 0.050 |  | 0.015 |  | 0.017 |  | 0.003 |  | 0.031 |  | 0.015 |  | 374.3 |  |
|  |  |  |  |  |  |  |  |  |  |  |  |  |  |  |  |  |  |
| **Within subjects** |  |  |  |  |  |  |  |  |  |  |  |  |  |  |  |  |  |
| Time | 3 | 0.884 | **15.43** | 0.121 | 39.70 | 0.543 | 31.32 | 0.349 | 70.60 | 0.168 | 21.94 | 0.792 | 44.16 | 0.762 | 62.36 | 262.1 | 1.43 |
| Time x CO_2_ | 6 | 0.103 | 1.789 | 0.047 | **15.50** | 0.071 | **4.10** | 0.073 | **14.83** | 0.079 | **10.29** | 0.134 | **7.48** | 0.263 | **21.54** | 1205.7 | **6.56** |
| Residual | 27 | 0.057 |  | 0.003 |  | 0.017 |  | 0.005 |  | 0.008 |  | 0.018 |  | 0.012 |  | 183.9 |  |
