# Supplementary material for: The Effect of CO2 on Algal Growth in Industrial Waste Water for Bioenergy and Bioremediation Applications
Source: PLoS One. 2013 Nov 22;8(11):e81631. doi: 10.1371/journal.pone.0081631 (PMC3838398; doi:10.1371/journal.pone.0081631)
Supplement: Table S3 — Repeated Measures Analyses of Variance of element removal rates from Ash Dam Water under different CO2 addition regimes. Significant main effects or interactions (P < 0.05) are highlighted in bold. (DOCX) [file pone.0081631.s004.docx]

Supporting Information

Table S3

|  |  | Al^a^ | | As | | B^a^ | | Cd^a^ | | Ni | | Se^a^ | | V^a^ | | Zn^a^ | |
| --- | --- | --- | --- | --- | --- | --- | --- | --- | --- | --- | --- | --- | --- | --- | --- | --- | --- |
| **Source** | **df** | **MS** | **F** | **MS** | **F** | **MS** | **F** | **MS** | **F** | **MS** | **F** | **MS** | **F** | **MS** | **F** | **MS** | **F** |
| **Between subjects** |  |  |  |  |  |  |  |  |  |  |  |  |  |  |  |  |  |
| CO_2_ | 2 | 1.699 | 1.49 | 0.011 | 4.15 | 0.946 | *25.07* | 0.089 | *5.72* | 0.007 | 2.176 | 0.110 | 0.44 | 0.418 | 1.394 | 0.029 | 0.083 |
| Residual | 9 | 0.143 |  | 0.003 |  | 0.038 |  | 0.016 |  | 0.003 |  | 0.250 |  | 0.3 |  | 0.342 |  |
|  |  |  |  |  |  |  |  |  |  |  |  |  |  |  |  |  |  |
| **Within subjects** |  |  |  |  |  |  |  |  |  |  |  |  |  |  |  |  |  |
| Time | 3 | 0.349 | *4.15* | 0.055 | *26.05* | 0.267 | *9.65* | 1.521 | *195.78* | 0.193 | *79.25* | 5.404 | **99.35** | 5.292 | **98.87** | 4.771 | **95.18** |
| Time x CO_2_ | 6 | 0.301 | **3.58** | 0.099 | **46.86** | 0.542 | **19.63** | 0.161 | **20.73** | 0.039 | **16.01** | 0.055 | 1.00 | 0.096 | 1.79 | 0.086 | 1.71 |
| Residual | 27 | 0.084 |  | 0.002 |  | 0.028 |  | 0.008 |  | 0.002 |  | 0.054 |  | 0.054 |  | 0.050 |  |
